# Macular vessel density in the superficial plexus is not associated to cerebrospinal fluid core biomarkers for Alzheimer’s disease in individuals with mild cognitive impairment: The NORFACE cohort

**DOI:** 10.3389/fnins.2023.1076177

**Published:** 2023-02-23

**Authors:** Marta Marquié, Ainhoa García-Sánchez, Emilio Alarcón-Martín, Joan Martínez, Miguel Castilla-Martí, Luis Castilla-Martí, Adelina Orellana, Laura Montrreal, Itziar de Rojas, Pablo García-González, Raquel Puerta, Clàudia Olivé, Amanda Cano, Isabel Hernández, Maitée Rosende-Roca, Liliana Vargas, Juan Pablo Tartari, Ester Esteban-De Antonio, Urszula Bojaryn, Mario Ricciardi, Diana M. Ariton, Vanesa Pytel, Montserrat Alegret, Gemma Ortega, Ana Espinosa, Alba Pérez-Cordón, Ángela Sanabria, Nathalia Muñoz, Núria Lleonart, Núria Aguilera, Lluís Tárraga, Sergi Valero, Agustín Ruiz, Mercè Boada

**Affiliations:** ^1^Ace Alzheimer Center Barcelona, Universitat Internacional de Catalunya (UIC), Barcelona, Spain; ^2^Networking Research Center on Neurodegenerative Diseases (CIBERNED), Instituto de Salud Carlos III, Madrid, Spain; ^3^Clínica Oftalmológica Dr. Castilla, Barcelona, Spain; ^4^Vista Alpina Eye Clinic, Visp, Switzerland; ^5^Ph.D. Programme in Surgery and Morphological Sciences, Universitat Autònoma de Barcelona, Barcelona, Spain; ^6^Hôpital Ophtalmique Jules-Gonin, Fondation Asile des Aveugles, University of Lausanne, Lausanne, Switzerland

**Keywords:** vessel density (VD), optical coherence tomography, Alzheimer, mild cognitive impairment, AT(N), cerebrospinal fluid, NORFACE

## Abstract

**Background:**

Optical coherence tomography angiography (OCT-A) is a novel method in the dementia field that allows the detection of retinal vascular changes. The comparison of OCT-A measures with established Alzheimer’s disease (AD)-related biomarkers is essential to validate the former as a marker of cerebrovascular impairment in the AD continuum. We aimed to investigate the association of macular vessel density (VD) in the superficial plexus quantified by OCT-A with the AT(N) classification based on cerebrospinal fluid (CSF) Aβ1-42, p181-tau and t-tau measurements in individuals with mild cognitive impairment (MCI).

**Materials and methods:**

Clinical, demographic, ophthalmological, OCT-A and CSF core biomarkers for AD data from the Neuro-ophthalmology Research at Fundació ACE (NORFACE) project were analyzed. Differences in macular VD in four quadrants (superior, nasal, inferior, and temporal) among three AT(N) groups [Normal, Alzheimer and Suspected non-Alzheimer pathology (SNAP)] were assessed in a multivariate regression model, adjusted for age, *APOE* ε4 status, hypertension, diabetes mellitus, dyslipidemia, heart disease, chronic obstructive pulmonary disease and smoking habit, using the Normal AT(N) group as the reference category.

**Results:**

The study cohort comprised 144 MCI participants: 66 Normal AT(N), 45 Alzheimer AT(N) and 33 SNAP AT(N). Regression analysis showed no significant association of the AT(N) groups with any of the regional macular VD measures (all, *p* > 0.16). The interaction between sex and AT(N) groups had no effect on differentiating VD. Lastly, CSF Aβ1-42, p181-tau and t-tau measures were not correlated to VD (all *r* < 0.13; *p* > 0.13).

**Discussion:**

Our study showed that macular VD measures were not associated with the AT(N) classification based on CSF biomarkers in patients with MCI, and did not differ between AD and other underlying causes of cognitive decline in our cohort.

## Introduction

Alzheimer’s disease (AD) is a slowly progressive neurodegenerative condition with long preclinical and prodromal stages that precede the onset of dementia. In this regard, the National Institute on Aging-Alzheimer’s Association (NIA-AA) research criteria for AD were reviewed in 2018 to introduce Aβ (A), tau (T) and neurodegeneration (N) biomarkers, resulting in the AT(N) framework (Jack et al., 2018). In these new criteria, the biological definition of AD, independently of the clinical syndrome, was established according to the underlying pathological processes occurring in the brain (amyloid plaque and neurofibrillary tangle deposition), which can be detected *in vivo* using fluid and/or neuroimaging biomarkers. Thus, individuals who are not yet demented [cognitively unimpaired or with mild cognitive impairment (MCI) (Albert et al., 2011)] can be then classified as presenting an underlying AD pathology if they show abnormal A and T biomarkers.

On this matter, the search for and validation of novel sensitive and specific biomarkers for the early detection of AD is currently a main research focus in the field, and includes plasma (Simrén et al., 2021), genomic (de Rojas et al., 2021), and retinal (Ngolab et al., 2019) parameters, among others. In particular, the retina is considered a “window into the brain” and can be accessed non-invasively through optical coherence tomography (OCT), which is a fast, inexpensive, non-invasive and widely accessible tool used for the diagnosis and monitoring of common ocular pathologies (Jaffe and Caprioli, 2004). The study of retinal biomarkers for AD started with the analysis of structural parameters (e.g., thickness and volume of the different retinal layers in the peripapillary and macular regions) and more recently, investigators have focused on the study of the retinal microvasculature [e.g., vessel density (VD) and size of the foveal avascular zone, mainly] (Jiang et al., 2018; Querques et al., 2019; Wu et al., 2020; Biscetti et al., 2021). Besides, the retinal microvascular network can be directly assessed *in vivo* using high-resolution OCT-angiography (OCT-A), while this cannot be done for cerebrovascular disease with brain imaging techniques. Cerebrovascular changes are a very common concomitant pathology to AD in elderly individuals with cognitive decline, as shown in autopsy studies (Gorelick et al., 2011; Attems and Jellinger, 2014), and are also involved in the pathophysiology of AD (Sweeney et al., 2019). It is hypothesized that the presence of retinal microvascular damage in patients with cognitive impairment could reflect microvascular damage occurring in the brain. Thus, OCT-A measures in the retina could be used as a proxy of brain vascular changes. Several publications have demonstrated abnormalities in retinal vascular parameters in AD and MCI patients compared to healthy controls, such as increases in the foveal avascular zone (FAZ) and decreases in VD (Cheung et al., 2014; Grewal et al., 2018; Jiang et al., 2018; Querques et al., 2019; Zabel et al., 2019; Zhang et al., 2019; Wu et al., 2020; Biscetti et al., 2021; Chalkias et al., 2021; Rifai et al., 2021), pointing to retinal vascular loss. The majority of cohorts reported in the literature, though, were relatively small and the results have not been validated in larger studies. A recent publication from our group, using data from a large clinical cohort, detected higher macular VD in the superficial plexus in MCI due to AD patients compared to cognitively healthy individuals (Marquié et al., 2022), contrary to previous literature results. Importantly, the inclusion of AD biomarkers in OCT-A studies in order to further validate this technique is warranted for the development of the field.

In the present study, we analyzed data from 144 individuals with MCI from the *Neuro Ophthalmology Research at Fundació ACE* (NORFACE) cohort with the goal to validate novel retinal vascular parameters against established AD core biomarkers. In particular, we investigated the association of macular VD in the superficial plexus quantified by OCT-A with the AT(N) classification based on cerebrospinal fluid (CSF) Aβ1-42, p181-tau and t-tau measurements. Our hypothesis was that MCI participants with an AD CSF profile (reduced Aβ and elevated tau) would show higher macular VD measures than those participants with a normal CSF profile, in line with the results of a recent publication from our group (Marquié et al., 2022).

## Materials and methods

### Study subjects

This study is embedded in the NORFACE project, which was founded in 2014 with the goal investigate retinal biomarkers of AD and analyze the relationship between retinal changes and different types of neurodegenerative disorders (Sánchez et al., 2018). Between February 2018 and March 2019, consecutive patients with a diagnosis of MCI (Petersen, 2004) evaluated at Ace Alzheimer Center Barcelona and who underwent, within 12 months, a lumbar puncture (LP) for the quantification of CSF core biomarkers for AD and an ophthalmological exam/OCT scan, were enrolled in the present study. Participants were recruited from three different sources: (1) the Memory Clinic, an outpatient diagnostic unit for individuals with cognitive decline referred by physicians from the Catalan Public Health System (Boada et al., 2014), (2) Fundació ACE’s Open House Initiative (Rodríguez-Gómez et al., 2015), a social program that assesses for free the cognitive status of individuals from the community without the need for a physician’s referral, and (3) the BIOFACE project, a research study of novel biomarkers in early onset MCI (Esteban-De Antonio et al., 2021). Inclusion criteria were: consensus-based clinical diagnosis of MCI (Petersen, 2004), age ≥50 years old, availability of *APOE* ε4 status, ability to complete the full ophthalmological exam and OCT scan and CSF core biomarkers for AD performed within 12 months of the OCT-A scan.

Further, a group of participants with subjective cognitive decline (SCD) from the Fundació ACE Healthy Brain Initiative (FACEHBI) cohort (Rodriguez-Gomez et al., 2017) with no objective impairment on formal cognitive testing and absence of brain amyloid uptake in a Florbetaben-PET scan (SCD Aβ-) were included as the control group (*n* = 83) in additional analyses. These SCD participants underwent the same cognitive testing, ophthalmological exam and OCT scan protocol than the MCI participants from the NORFACE cohort included in the main analyses.

### Clinical evaluation

Neurological, neuropsychological and social evaluations of the study participants were performed at Ace Alzheimer Center Barcelona. A consensus-based diagnosis of cognitive status was reached for each participant at the time of the study recruitment by a multidisciplinary team of professionals (Boada et al., 2014). Cognitive assessment consisted of the Spanish version of the Mini-Mental State Examination (MMSE) (Folstein et al., 1975; Blesa et al., 2001), the memory test of the Spanish version of the 7 min screening neurocognitive battery (del Ser Quijano et al., 2004), the Spanish version of the Neuropsychiatric Inventory Questionnaire (NPI-Q) (Cummings et al., 1994; Boada et al., 2005), the Geriatric Dementia Scale (GDS) (Reisberg et al., 1982), the Clinical Dementia Rating Score (CDR) (Morris, 1993), the Blessed Dementia Scale (Blessed et al., 1968), and a comprehensive neuropsychological battery of Fundació ACE (NBACE) (Alegret et al., 2012, 2013). Demographic information collected included age, gender and years of formal education. Past medical history collected included smoking habit (former or current), hypertension, diabetes mellitus, dyslipidemia, heart disease, and chronic obstructive pulmonary disease (COPD). This clinical information was obtained from participants’ medical records. MCI was defined using Petersen’s (Petersen, 2004) and the Cardiovascular health and cognition study criteria (Lopez et al., 2003).

### *APOE* genotyping

Genomic DNA was extracted from peripheral blood using the commercially available Chemagic system (Perkin Elmer). *APOE* genotypes were extracted from the Axiom SP array (Thermo Fisher Scientific) (Moreno-Grau et al., 2019; de Rojas et al., 2021). Ace Alzheimer Center Barcelona has this variable as a standard in its assessment protocols. Alternatively, the *APOE* genotypes were determined using fluorogenic allele-specific oligonucleotide probes (TaqMan assay; Life Technologies, Spain) for rs7412 (Test ID: C____904973_10) and rs429358 (Test ID: C___3084793_20). For the TaqMan assays, PCR and real-time fluorescence measurements were carried out on a QuantStudio3 real-time PCR system (Thermo Fisher Scientific, Spain) using the TaqMan Universal Master Mix (ref: 4364341, Life Technologies, Spain) methodology according to manufacturer’s instructions. The polymerase chain reaction was performed as follows: first, a pre-read step for 30 s at 60°C, denaturation for 10 min at 95°C, followed by 40 cycles at 95°C for 15 s and 60°C for 1 min, and a post read stage for 30 s at 60°C. The genotype was determined using the Genotyping App for Thermo Fisher Scientific Cloud by clustering analysis. The laboratory technicians were blinded to other study variables. *APOE* ε4 status (presence of at least one ε4 allele) was reported as a covariate in the analyses.

### Lumbar puncture and quantification of cerebrospinal fluid core biomarkers for Alzheimer’s disease

Lumbar punctures were performed at Ace Alzheimer Center Barcelona by an experienced neurologist under fasting conditions. CSF was collected passively in two 10-ml polypropylene tubes (Sarstedt Ref 62.610.018) centrifuged (2000 × g 10 min at 4°C), aliquoted and stored in polypropylene tubes (Sarstedt Ref 72.694.007) at −80°C until its use. Time delay between CSF collection and storage was less than 2 h. The collection protocol follows the recommendations of the Alzheimer’s Biomarkers Standardization Initiative (Vanderstichele et al., 2012). The day of the analysis, one aliquot of 0.5 mL was thawed and used for the determination of Aβ1-42, Total Tau (T-tau) and p181-tau. Aβ and tau proteins were quantified by either the commercially available enzyme linked immunosorbent assays (ELISA) (Innotest, Fujirebio Europe) (*n* = 114) or the chemiluminescence enzyme immunoassay (CLEIA) using the Lumipulse G 600 II automatic platform (Fujirebio Inc.) (Leitão et al., 2019) (*n* = 30).

Using CSF biomarkers, participants were classified into three categories according to the AT(N) scheme (Jack et al., 2018): Normal AD biomarkers (A-T-N-), Alzheimer’s continuum (including A+T-N-, A+T+N-, A+T+N+ and A+T-N+) and Non-AD pathologic changes [Suspected non-Alzheimer pathology (SNAP), including A-T+N-, A-T-N+ and A-T+N+], where A refers to aggregated Aβ, T to aggregated tau and N to neurodegeneration or neuronal injury (Supplementary Table 1). Cut-offs from the Ace Alzheimer Center Barcelona CSF program were used to dichotomize each CSF biomarker into ± as follows: for ELISA, Aβ1-42 < 676 pg/ml for A, p181-tau > 58 pg/ml for T and t-tau > 367 pg/ml for N; for CLEIA, Aβ1-42 < 796 pg/ml for A; p181-tau > 54 pg/ml for T and t-tau > 412 pg/ml for N (Orellana et al., 2022).

### Neuro-ophthalmological evaluation

In parallel to the cognitive assessment, study participants underwent a complete neuro-ophthalmological evaluation, which lasted about 20 min and was performed by an optometrist. The evaluation comprised: (1) a review of past ophthalmological diseases, treatments and surgeries, (2) monocular visual acuity assessment with the participants wearing their habitual correction for refractive error using a pinhole occluder and the Early Treatment of Diabetic Retinopathy Study (ETDRS) chart (Chew et al., 2009; Bokinni et al., 2015), (3) intraocular pressure (IOP) measurement by Icare tonometry (Pakrou et al., 2008), and (4) swept source (SS) OCT scan. More details can be found elsewhere (Marquié et al., 2022). The ophthalmologist and neurologists were blind to each other’s diagnosis.

### Optical coherence tomography and angiography

Participants were imaged with a DRI OCT Triton–Swept Source (SS) OCT (Topcon Co. Tokyo, Japan). The OCT exam was completed in about 5–10 min, no pupil dilation was required, and both eyes were scanned separately. The OCT Angiography Ratio Analysis (OCTARA) processing software was used to analyzed the data. An automatic segmentation method was employed to obtain measures of the superficial vascular plexus and the quantification of VD, expressed as the % of area covered by vessels. VD measures were obtained in a 6 mm × 6 mm area centered in the fovea, and the central area (1 mm circle) was excluded from the analysis. The parafoveal area, defined by two concentrical rings measuring 1 and 3 mm in diameter, respectively, was subdivided into four quadrants: nasal, superior, temporal and inferior. More details can be found in a recent publication from the NORFACE cohort (Marquié et al., 2022). The retinal vascular network consists of two separate beds: the superficial vascular plexus, at the level of the ganglion cell layer, and the deep vascular plexus, at the level of the outer plexiform layer (Toussaint et al., 1961). The FAZ is the region in the center of the macula within the fovea that is devoid of vasculature. In our analysis we used data from macular VD in the superficial vascular plexus.

Only VD measures from the right eye were used for the analysis, as in previous papers from our group (Sánchez et al., 2018, 2020; Marquié et al., 2020). OCT-related exclusion criteria were the following: lack of collaboration in the neuro-ophthalmological exam or OCT scan, OCT data obtained only from the left eye, presence of OCT artifacts and diseases that could affect retinal measurements [e.g., open-angle glaucoma and other neuropathies maculopathies, prior retinal surgery, intraocular pressure (IOP) ≥24 mmHg, high myopia (< −6 Dp) or hyperopia (> + 6 Dp) and optic nerve congenital abnormalities].

### Ethics statement

This study and its informed consent were approved by the ethics committees of the Hospital Clínic i Provincial de Barcelona in accordance with Spanish biomedical laws (Law 14/2007, July 3rd, about biomedical research; Royal Decree 1716/2011, November 18th) and followed the recommendations of the Declaration of Helsinki. All participants signed an additional informed consent for the lumbar puncture procedure.

### Statistical analysis

Data processing and analysis were carried out using R version 4.1.2 (R Core Team, n.d.).^1^ All data were examined for normality, skew and restriction of range. All quantitative variables were normally distributed. Frequency analysis and measures of central tendency and dispersion were used to describe the demographic (age, sex, education) and clinical (hypertension, diabetes mellitus, dyslipidemia, heart disease, chronic obstructive pulmonary disease (COPD) and smoking) variables among the three AT(N) groups (Normal, Alzheimer, and SNAP). To summarize the distribution of these demographic and clinical variables among the three AT(N) groups, bivariate Analysis of Variance (ANOVA) and Pearson’s chi-squared tests were executed.

For the final multivariate model, we included all clinical variables (hypertension, diabetes mellitus, dyslipidemia, heart disease, COPD, and smoking) as adjusting factors. To identify which demographic variables should be additionally included as adjusting factors in the final model, a multinomial regression analysis was executed to determine their differential distribution among the three AT(N) groups. An analysis was performed for the demographic variables (age, sex, education, and *APOE* ε4 status) including all the clinical variables (hypertension, diabetes mellitus, dyslipidemia, heart disease, COPD and smoking habit) as adjusting factors. The Normal AT(N) group was considered the reference category. For these analyses, alpha level was set at *p* < 0.05.

The main analyses consisted of four multivariate regression analyses, one for every macular VD measure (nasal, superior, temporal, and inferior quadrants), including the three AT(N) groups (Normal, Alzheimer and SNAP) as discriminant factors and adjusting their effect by all six clinical variables and those demographic factors that showed any significant effect in the former multinomial regression analysis. The Normal AT(N) group was considered the reference category. Regression coefficients (the mean change in the outcome variable for one unit of change in the predictor variable while holding other predictors in the model constant), betas (the degree of change in the outcome variable for every one unit of change in the predictor variable) and t (assessing whether the beta coefficient is significantly different from zero) are reported.

The former four multivariate regression analysis were rerun without including the A+T-N- participants within the AT(N) Alzheimer group (amyloidosis alone without tauopathy or neurodegeneration, *n* = 9).

Additionally, the former four multivariate regression analyses were repeated including a group of participants with subjective cognitive decline (SCD) and absent brain amyloid uptake in a FBB-PET scan (SCD Aβ-) from the Fundació ACE Healthy Brain Initiative (FACEHBI) cohort (Rodriguez-Gomez et al., 2017) as the reference category (*n* = 83).

For the former multivariate regression analyses, alpha level was set up at *p* < 0.004 (0.05/12) after Bonferroni’s correction for multiple comparisons.

The association between individual CSF biomarkers (Aβ1-42, p181-tau and t-tau) and each of the four macular VD measurements (nasal, superior, temporal and inferior quadrants) was explored using separate partial correlations, including the same covariates. These analyses were performed separately for the ELISA and CLEIA groups and also for the whole cohort after a log-transformation of each CSF biomarker value. For these correlation analyses, alpha level was set at *p* < 0.004 (0.05/12), after Bonferroni’s correction for multiple comparisons.

To investigate whether a differential effect could be detected when considering sex, the previous four multivariate regression analyses were executed again, including now the interaction between AT(N) group and sex as the main factor of interest and the same covariates. For these analyses, alpha level was set at *p* < 0.0125 (0.05/4), after Bonferroni’s correction for multiple comparisons.

## Results

### Demographic and clinical characteristics of the cohort

**FIGURE 1:**
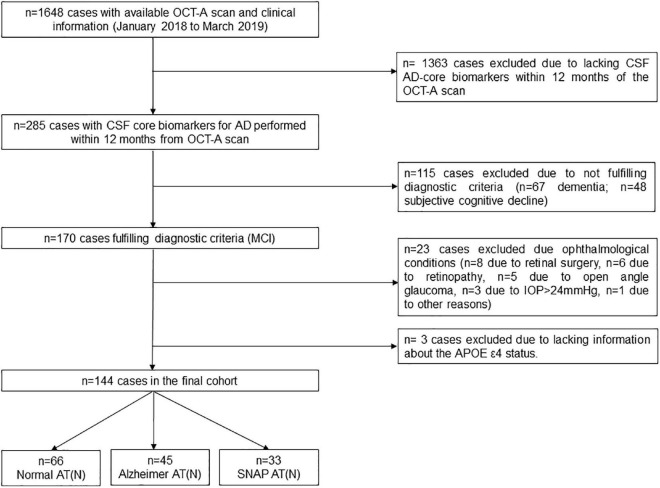
Participants’ selection algorithm. AD, Alzheimer’s disease; *APOE*, apolipoprotein E; OCT-A, optical coherence tomography-angiography; CSF, cerebrospinal fluid; IOP, intraocular pressure; MCI, mild cognitive impairment; SNAP, suspected non-Alzheimer pathology; VD, vessel density.

Data from 1,648 individuals with available clinical information and an OCT-A scan performed between January 2018 and March 2019 were initially reviewed (Figure 1). Several exclusion criteria were applied: lack of CSF AD core biomarkers data within 12 months of the OCT-A scan (*n* = 1363), not fulfilling the diagnostic criteria for MCI (*n* = 115), ophthalmological conditions that could interfere with OCT-A measurements (*n* = 23; *n* = 8 due to retinal surgery, *n* = 6 due to retinopathy, *n* = 5 due to open angle glaucoma, *n* = 3 due to IOP > 24 mmHg, *n* = 1 due to other reasons) and finally, lack of information on *APOE* ε4 status (*n* = 3).

The final sample consisted of 144 MCI individuals with available OCT-A scan and CSF AD core biomarkers performed within 12 months. Participants were classified into three groups according to the CSF AT(N) scheme (Jack et al., 2018): 66 Normal AT(N), 45 Alzheimer AT(N) and 33 SNAP AT(N). The demographic characteristics and past medical history of the cohort are displayed in Table 1.

**TABLE 1:** Demographic and clinical characteristics of the cohort.

	Total (*n* = 144)	Normal AT(N) (*n* = 66)	Alzheimer AT(N) (*n* = 45)	SNAP AT(N) (*n* = 33)	Intergroup significance
Age (years, mean ± SD)	70.10 ± 8.01	66.82 ± 8.52	73.02 ± 6.00	72.70 ± 6.98	<0.001^a^
Sex (% women)	57.64%	60.61%	46.67%	66.67%	0.169^b^
Education (years, mean ± SD)	8.17 ± 3.95	8.26 ± 3.23	8.64 ± 4.95	7.36 ± 3.73	0.395^a^
APOE ε4 status (% positive)	31.25%	18.18%	44.44%	39.39%	0.007^b^
Hypertension (%)	48.61%	42.42%	48.89%	60.61%	0.233^b^
Diabetes mellitus (%)	19.44%	18.18%	15.56%	27.27%	0.408^b^
Dyslipidemia (%)	38.89%	31.82%	40.00%	51.52%	0.163^b^
Heart disease (%)	9.72%	10.61%	6.67%	12.12%	0.728^b^
COPD (%)	14.58%	15.15%	11.11%	18.18%	0.642^b^
Smoking habit (%)	9.72%	12.12%	6.67%	9.09%	0.679^b^

Demographic and medical conditions among groups are summarized. ^a^1-factor ANOVA. ^b^Pearson’s Chi2 test. *APOE*, apolipoprotein E; COPD, chronic obstructive pulmonary disease; SD, standard deviation; SNAP, suspected non-Alzheimer pathology. Significance was set up at *p* < 0.05.

### Multinomial regression analysis of demographic characteristics among AT(N) groups

The multinomial regression analysis exploring the distribution of age, sex, education and the *APOE* ε4 status among the AT(N) groups, using the clinical variables (hypertension, diabetes mellitus, dyslipidemia, heart disease, COPD, smoking habit) as adjusting factors, showed that age and the *APOE* ε4 status had a significant effect, so both were included as adjusting factors in the final analysis (Supplementary Table 2).

### Multivariate regression analysis of macular VD differences among AT(N) groups

**FIGURE 2:**
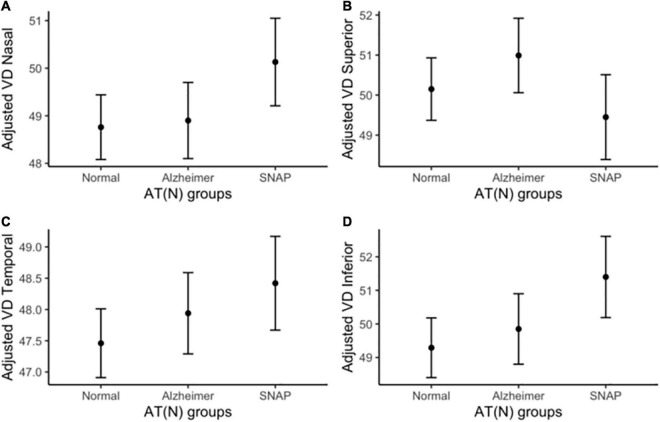
Adjusted macular VD measurements by AT(N) group. Macular VD differences among diagnostic groups in **(A)** nasal, **(B)** superior, **(C)** temporal, and **(D)** inferior quadrants. Macular VD measurements are adjusted by age, *APOE* ε4 status, hypertension, diabetes mellitus, dyslipidemia, heart disease, COPD and smoking habit. COPD, chronic obstructive pulmonary disease; n.s., non-significant; SNAP, suspected non-Alzheimer pathology; VD, vessel density.

Table 2 depicts the contribution of age, the *APOE* ε4 status, hypertension, diabetes mellitus, dyslipidemia, heart disease, COPD and smoking habit as adjusting factors and the AT(N) groups to macular VD variance in each quadrant. Regression models revealed no significant effect of AT(N) groups on VD values (all, *p* ≥ 0.162) (Figure 2). Similarly, *APOE* ε4 status, hypertension, diabetes mellitus, heart disease, COPD and smoking habit did not show any significant effect on VD (all, *p* ≥ 0.124). Age showed an inverse relationship with VD in the superior quadrant (*p* = 0.041) and dyslipidemia an inverse relationship with VD in the nasal quadrant (*p* = 0.043), but both were no longer significant after correction for multiple comparisons (*p* > 0.004).

**TABLE 2:** Multivariate regression analysis of macular VD measurements.

Covariates	Variables	Coefficient	t	Significance	Beta
Age	VD nasal	-0.09	-1.38	0.17	-0.13
	VD superior	-0.15	-2.06	0.041	-0.2
	VD temporal	-0.03	-0.53	0.596	-0.05
	VD inferior	-0.11	-1.32	0.189	-0.13
*APOE* ε4 status	VD nasal	0.36	0.37	0.71	0.03
	VD superior	0.86	0.76	0.446	0.07
	VD temporal	-0.01	-0.01	0.991	-0.01
	VD inferior	0.5	0.39	0.694	0.03
Hypertension	VD nasal	1.11	1.22	0.225	0.11
	VD superior	1.18	1.13	0.262	0.1
	VD temporal	0.84	1.14	0.258	0.1
	VD inferior	0.66	0.55	0.58	0.05
Diabetes mellitus	VD nasal	-0.56	-0.46	0.644	-0.04
	VD superior	-2.18	-1.55	0.124	-0.14
	VD temporal	-0.01	-0.01	0.991	-0.01
	VD inferior	0.2	0.13	0.9	0.01
Dyslipidemia	VD nasal	-1.95	-2.04	0.043	-0.19
	VD superior	0.47	0.43	0.67	0.04
	VD temporal	-1.49	-1.91	0.059	-0.18
	VD inferior	-1.54	-1.22	0.223	-0.11
Heart disease	VD nasal	2.02	1.33	0.186	0.12
	VD superior	1.26	0.72	0.475	0.06
	VD temporal	1.44	1.16	0.249	0.1
	VD inferior	-2.33	-1.17	0.246	-0.1
COPD	VD nasal	0.42	0.33	0.74	0.03
	VD superior	-0.2	-0.14	0.893	-0.01
	VD temporal	-0.68	-0.66	0.513	-0.06
	VD inferior	-1.95	-1.17	0.244	-0.1
Smoking habit	VD nasal	-0.62	-0.4	0.687	-0.04
	VD superior	0.64	0.36	0.718	0.03
	VD temporal	-0.74	-0.59	0.553	-0.05
	VD inferior	-2.84	-1.42	0.158	-0.12
AT(N) groups: Normal vs. Alzheimer	VD nasal	0.14	0.13	0.898	0.01
	VD superior	0.84	0.66	0.511	0.07
	VD temporal	0.48	0.53	0.596	0.05
	VD inferior	0.55	0.38	0.703	0.04
AT(N) groups: Normal vs. SNAP	VD nasal	1.37	1.16	0.248	0.11
	VD superior	-0.7	-0.52	0.608	-0.05
	VD temporal	0.96	0.99	0.322	0.1
	VD inferior	2.19	1.41	0.162	0.14

The multivariate regression analysis included the following adjusting factors: Age, *APOE* ε4 status, hypertension, diabetes mellitus, dyslipidemia, heart disease, COPD and smoking habit. *APOE*, apolipoprotein E; COPD, chronic obstructive pulmonary disease; SNAP, suspected non-Alzheimer pathology; VD, vessel density. Significance was set up at *p* < 0.004 after correcting for multiple comparisons.

Raw and adjusted sector-specific macular VD measures across diagnostic groups are displayed in Supplementary Table 3.

**FIGURE 3:**
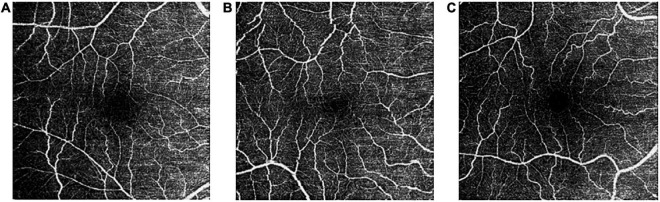
Representative macular VD images from the superficial vascular plexus for each AT(N) group: Normal **(A)**, Alzheimer **(B)**, and SNAP **(C)**. SNAP, suspected non-Alzheimer pathology; VD, vessel density.

Representative macular VD images from the superficial plexus for each AT(N) group are shown in Figure 3.

A sensitivity analysis was executed with the former multivariate regression analysis without including the A+T-N- participants within the Alzheimer AT(N) group (*n* = 9), showing similar results than the former, with no significant effect of AT(N) groups on VD values (Supplementary Table 4).

The former multivariate regression analysis were repeated using a cohort of 83 individuals with SCD with no evidence of brain amyloid deposition (negative FBB-PET scan) from the FACEHBI cohort as the reference category (SCD Aβ-), including age, sex, years of education, *APOE*ε4 status, hypertension, diabetes mellitus, dyslipidemia, heart disease, COPD and smoking habit as covariates. In this case, regression models revealed significant differences in VD in several macular regions between groups (see Supplementary Tables 5, 6). The Alzheimer AT(N) group showed higher VD measures in the temporal quadrant (*p* = 0.005) compared to the SCD Aβ- group, but that was no longer significant after correction for multiple comparisons (*p* > 0.004). The SNAP AT(N) group showed higher VD measures in the nasal (*p* = 0.008), temporal (*p* = 0.002) and inferior (*p* = 0.004) quadrants compared to the SCD Aβ- group, but only the temporal and the inferior quadrants remained significant after correction for multiple comparisons (*p* < 0.004). Additionally, age was negatively associated with VD in the superior quadrant (*p* = 0.004).

### Sex effect in macular VD across AT(N) groups

The interaction between sex and AT(N) groups had no effect in differentiating VD measurements, adjusted by age, age, *APOE* ε4 status, hypertension, diabetes mellitus, dyslipidemia, heart disease, COPD and smoking habit (Supplementary Table 7). Thus, differences in macular VD among AT(N) groups were not significantly influenced by sex.

### Association of macular VD with CSF Aβ, p-tau and t-tau measurements

Cerebrospinal fluid Aβ1-42, p-tau and t-tau values from the whole cohort (*n* = 144) were log-transformed and correlated separately with the four macular VD measures. None of the three CSF biomarkers showed a significant correlation with any macular VD measurements, adjusted by age, *APOE* ε4 status, hypertension, diabetes mellitus, dyslipidemia, heart disease, COPD, smoking habit and CSF technique (all *r* < 0.13; *p* ≥ 0.133) (see Table 3). Additionally, the analyses were repeated using raw CSF data for the ELISA (*n* = 114) and CLEIA (*n* = 30) groups separately, showing again a lack of correlation of each CSF biomarker with macular VD measures (see Supplementary Tables 8, 9).

**TABLE 3:** Partial correlation of macular VD and CSF measurements in the whole cohort.

CSF measurements (*n* = 144)	Variable	r	Significance
CSF Aβ1-42	VD nasal	0.02	0.808
	VD superior	-0.13	0.133
	VD temporal	-0.02	0.832
	VD inferior	0.06	0.499
CSF p181-tau	VD nasal	0.02	0.791
	VD superior	-0.06	0.516
	VD temporal	0.04	0.651
	VD inferior	0.12	0.154
CSF t-tau	VD nasal	0.04	0.676
	VD superior	-0.05	0.562
	VD temporal	0.03	0.767
	VD inferior	0.09	0.306

The model included the following adjusting factors: Age, *APOE* ε4 status, hypertension, diabetes mellitus, dyslipidemia, heart disease, COPD, smoking habit and CSF technique (ELISA vs. CLEIA). CSF biomarker data were log-transformed. Aβ, β-amyloid; *APOE*, apolipoprotein E; CLEIA, chemiluminescence enzyme immunoassay; COPD, chronic obstructive pulmonary disease; CSF, cerebrospinal fluid; ELISA, enzyme linked immunosorbent assays; VD, vessel density. Significance was set up at *p* < 0.004 after correcting for multiple comparisons.

## Discussion

In this study from the NORFACE cohort, we analyzed the relationship of macular VD in the superficial plexus and CSF AD core biomarkers in a cohort of 144 individuals with MCI. Our data showed that macular VD did not significantly differ among Normal, Alzheimer and SNAP AT(N) groups in individuals with MCI after adjustment for age, *APOE* ε4 status, hypertension, diabetes mellitus, dyslipidemia, heart disease, COPD and smoking habit. Additionally, macular VD was not associated to Aβ, p-tau and t-tau measures in CSF.

In a recent publication by our group, also analyzing macular VD measures from the NORFACE cohort (*n* = 672), patients clinically defined as MCI-Alzheimer (MCI-AD) and MCI-Vascular (MCI-Va) presented significant differences in macular VD, in opposite directions, in the temporal and inferior quadrants, respectively, compared to cognitively unimpaired (CU) individuals (Marquié et al., 2022). These results suggested that macular VD might be able to differentiate two pathogenic pathways (AD- and cerebrovascular-related) in the early stages of cognitive decline. In particular, MCI-AD participants showed higher VD in the temporal quadrant, while MCI-Va participants showed lower VD in the inferior quadrant compared to CU individuals. In the present work, we did not replicate these findings using a biological definition of AD in MCI patients [the Alzheimer AT(N) group], although it is worth noting that our reference category for comparison here was the Normal AT(N) MCI group, and not a CU group as in our previous article, and we did not include a clinical diagnosis of MCI-Vascular. We did, though, perform additional analysis using a cohort of 83 SCD Aβ- as the reference category, similarly to our previous publication (Marquié et al., 2022), and found that two of the AT(N) groups of MCI participants (Alzheimer and SNAP) showed significantly higher VD measured in several macular regions compared to the SCD Aβ- group, although only VD in the temporal and inferior quadrants from the SNAP group remained significant after correction for multiple comparisons. This result actually agrees with those of our previous publication, in which the clinically defined MCI-AD group showed higher VD in the temporal quadrant compared to CU individuals (Marquié et al., 2022). One potential explanation for the finding of higher macular VD in early stages of neurodegeneration [MCI individuals with Alzheimer and SNAP AT(N) profiles] is a temporary retinal vessel dilation and increased blood flow related to neuroinflammation, which takes place as a compensatory mechanism in response to the early vascular dysregulation (hypoxia) occurring in these diseases (Serrano-Pozo et al., 2011; Iturria-Medina et al., 2016; Sousa et al., 2018). These dilated retinal vessels would then become visible and cause a stronger vascular signal (higher VD) in OCT-A compared to controls.

Related to the latter and relevant to our results, the AT(N) classification (Jack et al., 2018) is restricted to Aβ (A), tau (T) and neurodegeneration (N) biomarkers, but does not take into account the presence of cerebrovascular pathology. As brain vascular lesions are very common in the elderly (Gorelick et al., 2011; Attems and Jellinger, 2014) and an important contributor to cognitive decline (Sweeney et al., 2019), potentially the three AT(N) groups of MCI patients depicted in our study (Normal, Alzheimer, and SNAP) could all have a similar burden of cerebrovascular lesions, and macular VD measures as quantified by OCT-A could be reflecting this. Unfortunately, in the present study, we did not have access to measures of brain vascular pathology from magnetic resonance imaging to compare them with VD.

Several other publications have investigated the association between retinal vascular parameters assessed by OCT-A and Alzheimer’s biomarkers quantified by either CSF or PET imaging in different stages of the AD continuum and found interesting results. O’Bryhim et al. (2018) reported increase in the size of the FAZ in preclinical AD participants (as defined by positive amyloid in CSF or PET) compared to cognitively normal controls. Frost et al. (2013) showed that cognitively healthy individuals from the AIBL study with PET-positive amyloid presented higher burden of retinal microvascular damage (larger venular branching asymmetry factor and arteriolar length-to-diameter ratio assessed in retinal photographs) compared to those with negative scans. Lee et al. (2020) showed decreased peripapillary capillary density in patients with subcortical vascular cognitive impairment compared to cognitively normal individuals and patients with PET-Aβ + cognitive impairment. In this study, capillary density was negatively associated with white matter volume quantified by brain MRI. In the other direction, van de Kreeke et al. (2019) reported a significantly higher VD in PET-Aβ + compared to PET-Aβ- healthy controls, but no differences in the FAZ. The authors attributed these results to increased retinal blood flow due to inflammation secondary to hypoxia in the preclinical AD stage, which allows microvessels normally not detected on OCT-A to become visible, thus resulting in a higher VD (van de Kreeke et al., 2019). Biscetti et al. (2021) reported a significant reduction in both vascular perfusion density and vessel length density in a group of 24 MCI with a CSF AD-profile compared to a group of 13 controls, while measures of fractal dimensions were higher in the MCI group. The authors claimed that vascular perfusion density might reflect amyloid angiopathy-related chronic injury, while fractal dimensions could show early vessel recruitment as a compensatory mechanism (Biscetti et al., 2021). Lastly, Elahi et al. (2021) investigated a cohort of 75 cognitively normal adults, showing significantly reduced macular capillary density in *APOE* ε4 carriers compared to non-carriers, while no differences were detected between PET-Aβ + vs. PET-Aβ–individuals.

An additional finding of our study was a negative correlation between age and macular VD in the superior quadrant (although no longer significant after correcting for multiple comparisons), meaning that the elder individuals in our cohort showed lower VD in this region. Similarly, previous publications from the NORFACE cohort showed an association of age with several retinal measures (Sánchez et al., 2018, 2020). Other studies have also demonstrated a decrease in retinal vascular measures, including VD, with aging (Barteselli et al., 2012; Yu et al., 2015; Iafe et al., 2016; Wei et al., 2017). Related to this issue, the concept of “retinal age” has recently emerged. Several articles have detected, by analyzing retinal fundus images from large cohorts using deep learning algorithms, that the retinal age gap (the difference between retinal age and chronological age) is a robust biomarker for aging and closely related to the risk of mortality (Nusinovici et al., 2022; Zhu et al., 2022b). Moreover, it is associated with arterial stiffness and future cardiovascular disease events (Zhu et al., 2022a) and is able to identify individuals at high risk of developing future Parkinson’s disease (Hu et al., 2022). These findings suggest that retinal measures could potentially be used as a proxy of biological aging, but further research in this area is needed.

Lastly, our data showed no effects of the interaction between sex and the AT(N) group in predicting macular VD. Although sex is usually added as a covariate in analyses, few publications investigated sex as an independent factor potentially involved in OCT-A measures. Hashmani et al., showed that males had greater VD than females in the foveal region (Hashmani et al., 2019), while Su et al. (2022) also described sex-dependent differences in the effect of aging on retinal VD changes. The effect of sex on FAZ size remains uncertain, with discrepant results among studies (Samara et al., 2015; Yu et al., 2015; Tan et al., 2016).

We acknowledge that our study had several limitations. First, the lack of significant results could be related to the small sample size of the AT(N) groups. Second, the results were cross-sectional, and thus not able to show longitudinal changes in diagnosis, macular VD and CSF Aβ and tau measures. Third, we did not use a cognitively healthy cohort as a control group for the comparisons, but instead MCI patients with a Normal AT(N) profile in the CSF, who could potentially harbor a certain burden of cerebrovascular pathology. Fourth, no MRI measures of brain vascular pathology were available to compare with the retinal vascular pathology. Lastly, the VD measurements were limited to the macular region and the superficial vascular plexus, and we lacked information about changes in the FAZ and VD in the deep plexus.

We also consider that our study had several strengths compared to previous works. Our cohort consisted of a large and single-site sample of MCI patients with OCT-A and CSF AD core biomarkers performed within 12 months. We used age, *APOE* ε4 status and cardiovascular conditions as covariates in all our analyses. Lastly, the neurologist and optometrist were blinded to each other’s diagnosis.

In summary, our study detected no significant differences on a retinal vascular measure (macular VD in the superficial vascular plexus) among Normal, Alzheimer and SNAP AT(N) groups in a cohort of MCI patients, and a lack of association with Aβ and tau measures in CSF.

## Data availability statement

According Ace Alzheimer Center Barcelona’s policies and following the Spanish Law 14/2007 of 3 July on Biomedical Research, clinical data will not be uploaded to servers or public repositories. The datasets generated and/or analyzed for this study will be made available by the corresponding author on reasonable request, after internal Data Accessing Committee evaluation, Ethics Committee clearance and MDTA signature between institutions.

## Ethics statement

This study and its informed consent were approved by the Ethics Committees of the Hospital Clínic i Provincial de Barcelona in accordance with Spanish biomedical laws (Law 14/2007, July 3rd, about biomedical research; Royal Decree 1716/2011, November 18th) and followed the recommendations of the Declaration of Helsinki. All participants signed an additional informed consent for the Lumbar puncture procedure. The patients/participants provided their written informed consent to participate in this study.

## Author contributions

MM, LT, AR, and MB were involved in the study design and conduct. MM, AG-S, SV, MC-M, and LC-M were in charge of the data analysis. MM wrote the manuscript. MM, AG-S, and SV performed the statistical analysis. EA-M, AG-S, and IR prepared the databases. MM, JM, IH, MR-R, LV, JPT, EE-D, UB, MA, GO, AE, AP-C, ÁS, NA, AO, and LM were involved in the acquisition of data. All authors contributed to the interpretation of findings, critical review of the manuscript, approval of the final manuscript, and agreement to be accountable for all aspects of the work.

## Footnotes

1
https://www.R-project.org/
