# Supplementary material for: Macular vessel density in the superficial plexus is not associated to cerebrospinal fluid core biomarkers for Alzheimer’s disease in individuals with mild cognitive impairment: The NORFACE cohort
Source: Front Neurosci. 2023 Feb 23;17:1076177. doi: 10.3389/fnins.2023.1076177 (PMC9995931; doi:10.3389/fnins.2023.1076177)
Supplement: Supplementary file 1 [file Data_Sheet_1.docx]

**Supplementary Information**

**Supplementary Table S1. AT(N) groups**

| **AT(N) groups** | **CSF Aβ1-42** | **CSF p181-tau** | **CSF t-tau** |
| --- | --- | --- | --- |
| Normal | **-** | **-** | **-** |
| Alzheimer continuum | **+** | **-** | **-** |
|  | **+** | **+** | **-** |
|  | **+** | **-** | **+** |
|  | **+** | **+** | **+** |
| SNAP | **-** | **+** | **-** |
|  | **-** | **-** | **+** |
|  | **-** | **+** | **+** |

Abbreviations: CSF = cerebrospinal fluid; SNAP = suspected non-Alzheimer pathology.

**Supplementary Table S2. Multinomial regression analysis of the distribution of age, sex, education and APOE ε4 status among AT(N) groups**

| **AT(N) groups comparisons** | **Variables** | **OR** | **95% confidence interval** | **Standard error** | **Significance** |
| --- | --- | --- | --- | --- | --- |
| Normal AT(N) vs Alzheimer AT(N) | Age | 1.13 | 1.06 - 1.21 | 0.04 | <0.001* |
|  | Sex | 0.67 | 0.26 - 1.69 | 0.32 | 0.393 |
|  | Education | 1.04 | 0.93 - 1.16 | 0.06 | 0.480 |
|  | *APOE* ε4 status | 3.71 | 1.45 - 9.50 | 1.78 | 0.006* |
| Normal AT(N) vs SNAP AT(N) | Age | 1.12 | 1.04 - 1.20 | 0.04 | 0.001* |
|  | Sex | 1.88 | 0.65 – 5.42 | 1.02 | 0.242 |
|  | Education | 0.98 | 0.87 - 1.10 | 0.06 | 0.683 |
|  | *APOE* ε4 status | 2.91 | 1.05 - 8.09 | 1.52 | 0.041* |

The multinomial regression analysis included the following adjusting factors: hypertension, diabetes mellitus, dyslipidemia, heart disease, COPD and smoking habit.

Abbreviations: *APOE* = apolipoprotein E; COPD = chronic obstructive pulmonary disease; OR = Odds Ratio; SNAP = suspected non-Alzheimer pathology.

Significance was set up at p<0.05.

**Supplementary Table S3. Raw and adjusted macular VD differences across AT(N) groups**

| **Group (n)** | **Mean** | **SD** | **Mean^aa^** | **SEM^aa^** |
| --- | --- | --- | --- | --- |
| **VD Nasal** | | | | |
| Normal AT(N) (n=66) | 49.08 | 5.04 | 48.76 | 0.68 |
| Alzheimer AT(N) (n=45) | 48.64 | 5.44 | 48.90 | 0.80 |
| SNAP AT(N) (n=33) | 49.85 | 4.95 | 50.13 | 0.92 |
| **VD Superior** | | | | |
| Normal AT(N) (n=66) | 50.47 | 6.02 | 50.15 | 0.78 |
| Alzheimer AT(N) (n=45) | 50.71 | 4.22 | 50.99 | 0.93 |
| SNAP AT(N) (n=33) | 49.18 | 7.69 | 49.45 | 1.06 |
| **VD Temporal** | | | | |
| Normal AT(N) (n=66) | 47.59 | 4.20 | 47.46 | 0.55 |
| Alzheimer AT(N) (n=45) | 47.84 | 3.87 | 47.94 | 0.65 |
| SNAP AT(N) (n=33) | 48.27 | 4.49 | 48.42 | 0.75 |
| **VD Inferior** | | | | |
| Normal AT(N) (n=66) | 49.55 | 6.54 | 49.29 | 0.89 |
| Alzheimer AT(N) (n=45) | 49.80 | 6.21 | 49.85 | 1.05 |
| SNAP AT(N) (n=33) | 51.03 | 7.95 | 51.48 | 1.21 |

Raw and adjusted macular VD means, standard deviation (SD) and standard error of the mean (SEM) are shown. Dispersion is shown as SEM. Statistical significance was set up at p<0.05.

^aa^ = after adjustment for the following factors: age, *APOE* ε4 status, hypertension, diabetes mellitus, dyslipidemia, heart disease, COPD and smoking habit.

Abbreviations: *APOE* = apolipoprotein E; COPD = chronic obstructive pulmonary disease; SD = standard deviation; SEM = standard error of the mean; SNAP = suspected non-Alzheimer pathology; VD = vessel density.

**Supplementary Table S4. Multivariate regression analysis of macular VD measurements without including A+T-N- participants (n=9) within the Alzheimer AT(N) group.**

| Covariates | Variables | Coefficient | t | Significance | Beta |
| --- | --- | --- | --- | --- | --- |
| Age | VD Nasal | -0.10 | -1.52 | 0.132 | -0.15 |
|  | VD Superior | -0.15 | -2.01 | 0.046 | -0.20 |
|  | VD Temporal | -0.02 | -0.45 | 0.652 | -0.05 |
|  | VD Inferior | -0.13 | -1.55 | 0.123 | -0.16 |
| *APOE* ε4 status | VD Nasal | 0.17 | 0.16 | 0.872 | 0.01 |
|  | VD Superior | 1.08 | 0.93 | 0.355 | 0.08 |
|  | VD Temporal | -0.20 | -0.25 | 0.804 | -0.02 |
|  | VD Inferior | 0.78 | 0.57 | 0.567 | 0.05 |
| Hypertension | VD Nasal | 1.48 | 1.54 | 0.126 | 0.14 |
|  | VD Superior | 1.51 | 1.39 | 0.168 | 0.13 |
|  | VD Temporal | 1.24 | 1.62 | 0.108 | 0.15 |
|  | VD Inferior | 0.82 | 0.65 | 0.516 | 0.06 |
| Diabetes mellitus | VD Nasal | -0.61 | -0.47 | 0.638 | -0.05 |
|  | VD Superior | -2.93 | -2.01 | 0.047 | -0.19 |
|  | VD Temporal | -0.34 | -0.34 | 0.737 | -0.03 |
|  | VD Inferior | 0.11 | 0.07 | 0.947 | 0.01 |
| Dyslipidemia | VD Nasal | -2.08 | -2.06 | 0.041 | -0.19 |
|  | VD Superior | 0.70 | 0.61 | 0.543 | 0.06 |
|  | VD Temporal | -1.64 | -2.05 | 0.043 | -0.19 |
|  | VD Inferior | -1.62 | -1.22 | 0.225 | -0.11 |
| Heart disease | VD Nasal | 1.92 | 1.24 | 0.218 | 0.11 |
|  | VD Superior | 1.39 | 0.79 | 0.431 | 0.07 |
|  | VD Temporal | 1.46 | 1.18 | 0.239 | 0.11 |
|  | VD Inferior | -2.37 | -1.16 | 0.249 | -0.10 |
| COPD | VD Nasal | 0.43 | 0.33 | 0.739 | 0.03 |
|  | VD Superior | -0.13 | -0.09 | 0.930 | -0.01 |
|  | VD Temporal | -0.60 | -0.58 | 0.560 | -0.05 |
|  | VD Inferior | -2.01 | -1.18 | 0.239 | -0.11 |
| Smoking | VD Nasal | -0.84 | -0.54 | 0.591 | -0.05 |
|  | VD Superior | 0.67 | 0.38 | 0.707 | 0.03 |
|  | VD Temporal | -0.77 | -0.62 | 0.537 | -0.06 |
|  | VD Inferior | -3.07 | -1.49 | 0.138 | -0.14 |
| AT(N) groups: Normal AT(N) vs Alzheimer AT(N) | VD Nasal | 0.55 | 0.46 | 0.648 | 0.05 |
|  | VD Superior | 0.40 | 0.30 | 0.768 | 0.03 |
|  | VD Temporal | 0.32 | 0.33 | 0.741 | 0.03 |
|  | VD Inferior | 1.17 | 0.74 | 0.464 | 0.07 |
| AT(N) groups: Normal AT(N) vs SNAP AT(N) | VD Nasal | 1.45 | 1.20 | 0.232 | 0.12 |
|  | VD Superior | -0.78 | -0.57 | 0.568 | -0.06 |
|  | VD Temporal | 0.97 | 1.01 | 0.317 | 0.10 |
|  | VD Inferior | 2.27 | 1.43 | 0.156 | 0.14 |

The multivariate regression analysis included the following adjusting factors: age, *APOE* ε4 status, hypertension, diabetes mellitus, dyslipidemia, heart disease, COPD and smoking habit.

Abbreviations: *APOE* = apolipoprotein E; COPD = chronic obstructive pulmonary disease; SNAP = suspected non-Alzheimer pathology; VD = vessel density.

Significance was set up at p<0.004 after correcting for multiple comparisons.

**Supplementary Table S5. Multivariate regression analysis of macular VD measurements with a control group of SCD Aβ- individuals**

| Covariates | Variables | Coefficient | t | Significance | Beta |
| --- | --- | --- | --- | --- | --- |
| Age | VD Nasal | -0.10 | -2.22 | 0.028 | -0.16 |
|  | VD Superior | -0.14 | -2.90 | 0.004* | -0.21 |
|  | VD Temporal | -0.05 | -1.44 | 0.152 | -0.10 |
|  | VD Inferior | -0.08 | -1.40 | 0.163 | -0.10 |
| Sex | VD Nasal | 0.47 | 0.68 | 0.500 | 0.05 |
|  | VD Superior | -0.24 | -0.30 | 0.763 | -0.02 |
|  | VD Temporal | -0.41 | -0.71 | 0.478 | -0.05 |
|  | VD Inferior | 0.97 | 1.10 | 0.274 | 0.08 |
| Years of education | VD Nasal | -0.05 | -0.66 | 0.509 | -0.06 |
|  | VD Superior | -0.17 | -1.97 | 0.050 | -0.18 |
|  | VD Temporal | -0.08 | -1.25 | 0.214 | -0.11 |
|  | VD Inferior | 0.04 | 0.45 | 0.656 | 0.04 |
| *APOE* ε4 status | VD Nasal | 0.40 | 0.55 | 0.585 | 0.04 |
|  | VD Superior | 0.99 | 1.20 | 0.231 | 0.08 |
|  | VD Temporal | 0.15 | 0.24 | 0.808 | 0.02 |
|  | VD Inferior | 0.49 | 0.53 | 0.598 | 0.04 |
| Hypertension | VD Nasal | 0.80 | 1.21 | 0.229 | 0.08 |
|  | VD Superior | 0.70 | 0.95 | 0.345 | 0.07 |
|  | VD Temporal | 0.09 | 0.16 | 0.872 | 0.01 |
|  | VD Inferior | 0.46 | 0.55 | 0.581 | 0.04 |
| Diabetes mellitus | VD Nasal | -0.98 | -1.01 | 0.314 | -0.07 |
|  | VD Superior | -1.95 | -1.78 | 0.076 | -0.13 |
|  | VD Temporal | -0.10 | -0.12 | 0.902 | -0.01 |
|  | VD Inferior | -0.67 | -0.55 | 0.587 | -0.04 |
| Dyslipidemia | VD Nasal | -1.21 | -1.78 | 0.077 | -0.12 |
|  | VD Superior | 0.47 | 0.62 | 0.536 | 0.04 |
|  | VD Temporal | -0.99 | -1.77 | 0.078 | -0.12 |
|  | VD Inferior | -1.08 | -1.26 | 0.210 | -0.09 |
| Heart disease | VD Nasal | 1.94 | 1.85 | 0.066 | 0.13 |
|  | VD Superior | 1.39 | 1.18 | 0.240 | 0.08 |
|  | VD Temporal | 1.20 | 1.38 | 0.169 | 0.09 |
|  | VD Inferior | -0.44 | -0.33 | 0.739 | -0.02 |
| COPD | VD Nasal | -0.15 | -0.17 | 0.864 | -0.01 |
|  | VD Superior | 0.20 | 0.21 | 0.833 | 0.01 |
|  | VD Temporal | -0.78 | -1.11 | 0.270 | -0.07 |
|  | VD Inferior | -0.99 | -0.91 | 0.366 | -0.06 |
| Smoking | VD Nasal | -0.58 | -0.64 | 0.525 | -0.04 |
|  | VD Superior | 0.91 | 0.88 | 0.378 | 0.06 |
|  | VD Temporal | -0.50 | -0.66 | 0.510 | -0.04 |
|  | VD Inferior | -0.61 | -0.52 | 0.603 | -0.04 |
| Groups:  SCD Aβ- vs Normal AT(N) | VD Nasal | 1.80 | 1.86 | 0.065 | 0.17 |
|  | VD Superior | 0.89 | 0.82 | 0.415 | 0.08 |
|  | VD Temporal | 1.90 | 2.37 | 0.019 | 0.21 |
|  | VD Inferior | 2.34 | 1.89 | 0.060 | 0.18 |
| Groups:  SCD Aβ- vs Alzheimer AT(N) | VD Nasal | 2.01 | 1.89 | 0.060 | 0.17 |
|  | VD Superior | 1.77 | 1.48 | 0.141 | 0.13 |
|  | VD Temporal | 2.47 | 2.82 | 0.005 | 0.24 |
|  | VD Inferior | 3.01 | 2.23 | 0.027 | 0.20 |
| Groups:  SCD Aβ- vs SNAP AT(N) | VD Nasal | 3.12 | 2.68 | 0.008 | 0.23 |
|  | VD Superior | 0.05 | 0.04 | 0.972 | 0.01 |
|  | VD Temporal | 2.98 | 3.10 | 0.002* | 0.26 |
|  | VD Inferior | 4.36 | 2.94 | 0.004* | 0.26 |

The multivariate regression analysis included the following adjusting factors: age, sex, years of education, *APOE* ε4 status, hypertension, diabetes mellitus, dyslipidemia, heart disease, COPD and smoking habit.

Abbreviations: Aβ = β-amyloid; *APOE* = apolipoprotein E; COPD = chronic obstructive pulmonary disease; SCD = subjective cognitive decline; SNAP = suspected non-Alzheimer pathology; VD = vessel density.

Significance was set up at p<0.004 after correcting for multiple comparisons.

**Supplementary Table S6.** **Raw and adjusted macular VD differences across SCD Aβ- and AT(N) groups**

| **Group (n)** | **Mean** | **SD** | **Mean^aa^** | **SEM^aa^** |
| --- | --- | --- | --- | --- |
| **VD Nasal** | | | | |
| SCD Aβ- (n=83) | 46.75 | 3.54 | 46.88 | 0.63 |
| Normal AT(N) (n=66) | 49.08 | 5.04 | 48.68 | 0.62 |
| Alzheimer AT(N) (n=45) | 48.64 | 5.44 | 48.88 | 0.74 |
| SNAP AT(N) (n=33) | 49.85 | 4.95 | 50.00 | 0.86 |
| **VD Superior** | | | | |
| SCD Aβ- (n=83) | 48.47 | 3.84 | 48.98 | 0.71 |
| Normal AT(N) (n=66) | 50.47 | 6.02 | 49.88 | 0.70 |
| Alzheimer AT(N) (n=45) | 50.71 | 4.22 | 50.75 | 0.84 |
| SNAP AT(N) (n=33) | 49.18 | 7.69 | 49.03 | 0.97 |
| **VD Temporal** | | | | |
| SCD Aβ- (n=83) | 44.87 | 3.01 | 45.27 | 0.52 |
| Normal AT(N) (n=66) | 47.59 | 4.20 | 47.17 | 0.51 |
| Alzheimer AT(N) (n=45) | 47.84 | 3.87 | 47.74 | 0.61 |
| SNAP AT(N) (n=33) | 48.27 | 4.49 | 48.25 | 0.71 |
| **VD Inferior** | | | | |
| SCD Aβ- (n=83) | 47.37 | 3.62 | 47.10 | 0.81 |
| Normal AT(N) (n=66) | 49.55 | 6.54 | 49.44 | 0.79 |
| Alzheimer AT(N) (n=45) | 49.80 | 6.21 | 50.12 | 0.95 |
| SNAP AT(N) (n=33) | 51.03 | 7.95 | 51.47 | 1.10 |

Raw and adjusted macular VD means, standard deviation (SD) and standard error of the mean (SEM) are shown. Dispersion is shown as SEM. Statistical significance was set up at p<0.05.

^aa^ = after adjustment for the following factors: age, sex, years of education, *APOE* ε4 status, hypertension, diabetes mellitus, dyslipidemia, heart disease, COPD and smoking habit.

Abbreviations: Aβ = β-amyloid; *APOE* = apolipoprotein E; COPD = chronic obstructive pulmonary disease; SCD = subjective cognitive decline; SNAP = suspected non-Alzheimer pathology; VD = vessel density.

**Supplementary Table S7. Multivariate regression analysis of the interaction of sex and AT(N) profiles in predicting macular VD measurements**

| **AT(N) group comparisons** | **Variables** | **Coefficient** | **t** | **Significance** | **Beta** |
| --- | --- | --- | --- | --- | --- |
| Interaction sex x AT(N) profiles (Normal AT(N) vs Alzheimer AT(N)) | VD Nasal | 3.50 | 1.70 | 0.093 | 0.24 |
|  | VD Superior | 3.03 | 1.26 | 0.210 | 0.18 |
|  | VD Temporal | 2.97 | 1.76 | 0.081 | 0.25 |
|  | VD Inferior | -1.55 | -0.57 | 0.573 | -0.08 |
| Interaction sex x AT(N) profiles (Normal AT(N) vs SNAP AT(N)) | VD Nasal | 2.22 | 0.93 | 0.352 | 0.16 |
|  | VD Superior | 2.66 | 0.96 | 0.339 | 0.16 |
|  | VD Temporal | 1.84 | 0.95 | 0.346 | 0.16 |
|  | VD Inferior | -1.81 | -0.57 | 0.569 | -0.10 |

The multivariate regression analysis included the following adjusting factors: age, *APOE* ε4 status, hypertension, diabetes mellitus, dyslipidemia, heart disease, COPD and smoking habit.

Abbreviations: *APOE* = apolipoprotein E; COPD = chronic obstructive pulmonary disease; SNAP = suspected non-Alzheimer pathology; VD = vessel density.

Significance was set up at p<0.0125 after correcting for multiple comparisons.

**Supplementary Table S8. Partial correlation of macular VD with CSF measurements (using ELISA)**

| CSF measurements (n=114) | Variable | r | Significance |
| --- | --- | --- | --- |
| CSF Aβ1-42 | VD Nasal | -0.10 | 0.330 |
|  | VD Superior | -0.12 | 0.209 |
|  | VD Temporal | 0.07 | 0.487 |
|  | VD Inferior | 0.11 | 0.253 |
| CSF p181-tau | VD Nasal | 0.02 | 0.879 |
|  | VD Superior | -0.07 | 0.498 |
|  | VD Temporal | 0.02 | 0.859 |
|  | VD Inferior | 0.05 | 0.619 |
| CSF t-tau | VD Nasal | 0.03 | 0.758 |
|  | VD Superior | -0.03 | 0.754 |
|  | VD Temporal | -0.01 | 0.992 |
|  | VD Inferior | 0.03 | 0.780 |

The model included the following adjusting factors: age, *APOE* ε4 status, hypertension, diabetes mellitus, dyslipidemia, heart disease, COPD and smoking habit.

Abbreviations: Aβ = β-amyloid; *APOE* = apolipoprotein E; COPD = chronic obstructive pulmonary disease; CSF = cerebrospinal fluid; ELISA = enzyme linked immunosorbent assays; VD = vessel density.

Significance was set up at p<0.004 after correcting for multiple comparisons.

**Supplementary Table S9. Partial correlation of macular VD with CSF measurements (using CLEIA)**

| CSF measurements (n=30) | Variable | r | Significance |
| --- | --- | --- | --- |
| CSF Aβ1-42 | VD Nasal | 0.41 | 0.083 |
|  | VD Superior | -0.23 | 0.343 |
|  | VD Temporal | -0.13 | 0.596 |
|  | VD Inferior | -0.18 | 0.465 |
| CSF p181-tau | VD Nasal | -0.30 | 0.211 |
|  | VD Superior | -0.21 | 0.382 |
|  | VD Temporal | 0.04 | 0.867 |
|  | VD Inferior | 0.36 | 0.134 |
| CSF t-tau | VD Nasal | -0.33 | 0.164 |
|  | VD Superior | -0.29 | 0.235 |
|  | VD Temporal | 0.03 | 0.897 |
|  | VD Inferior | 0.29 | 0.222 |

The model included the following adjusting factors: age, APOE ε4 status, hypertension, diabetes mellitus, dyslipidemia, heart disease, COPD and smoking habit.

Abbreviations: Aβ = β-amyloid; *APOE* = apolipoprotein E; CLEIA = chemiluminescense enzyme immunoassay; COPD = chronic obstructive pulmonary disease; CSF = cerebrospinal fluid; VD = vessel density.

Significance was set up at p<0.004 after correcting for multiple comparisons.
